# Influence of exposure scenario on the sensitivity to caffeine

**DOI:** 10.1007/s11356-023-30945-3

**Published:** 2023-11-17

**Authors:** Niedja Santos, Miguel Oliveira, Inês Domingues

**Affiliations:** Centre for Environmental and Marine Studies (CESAM), Department of Biology, University of Aveiro, Campus Universitário de Santiago, 3810-193 Aveiro, Portugal

**Keywords:** Coffee, Life stage, Embryotoxicity, Chorion, Exposure window, Recovery

## Abstract

The chorion acts as a protective barrier, restricting some chemical absorption into the embryo and the surrounding fluids. In this sense, larvae may only have direct contact with some chemicals after dechorionation. This study aimed to evaluate the effects of caffeine (CAF) (0, 13, 20, 44, 67, and 100 mg.L^−1^) under different exposure scenarios (embryos with chorion or embryos/larvae already hatched) and rank the stage sensitivity. Thus, three scenarios were investigated: from 2 to 120 hours post fertilization (hpf) (5 days of exposure- 5dE), from 72 to 120 hpf (2dE), and from 96 to 120 hpf (1dE). Heart rate (48 hpf) and energy reserves (120 hpf) were measured in the 5dE scenario, and behavior and acetylcholinesterase (AChE) activity were evaluated at 120 hpf in all scenarios (5dE, 2dE, and 1dE). At 120 hpf, some of the fish was transferred to clean medium for a 10 days depuration period (10dPE). Behavior and AChE activity were assessed after this period. In the 5dE scenario, CAF increased heartbeat (13, 20, and 30 mg.L^−1^) and reduced carbohydrates (67, and 100 mg.L^−1^), while inhibiting AChE activity (100 mg.L^−1^) in the 5dE, 2dE, and 1dE scenarios. CAF reduced the total distance moved in the 5dE (67, and 100 mg.L^−1^), 2dE (20, 30, 44, 67, and 100 mg.L^−1^), and 1dE fish (67, and 100 mg.L^−1^) and increased erratic movements. Based on the lowest observed effect concentration (LOEC) for total distance moved (20 mg.L^−1^) and higher inhibition of AChE activity (100 mg.L^−1^) (65%), 2dE fish appear to be more sensitive to CAF. After 10dPE, a recovery in behavior was detected in all scenarios (5dE, 2dE, and 1dE). AChE activity remained inhibited in the 2dE scenario while increasing in the 1dE scenario. This study demonstrated that the presence of the chorion is an important factor for the analysis of CAF toxicity. After the loss of the chorion, organisms show greater sensitivity to CAF and can be used to evaluate the toxicity of various substances, including nanomaterials or chemicals with low capacity to cross the chorion. Therefore, the use of hatched embryos in toxicity tests is suggested, as they allow a shorter and less expensive exposure scenario that provides similar outcome as the conventional scenario.

**Supplementary Information:**

The online version contains supplementary material available at 10.1007/s11356-023-30945-3.

## Introduction

The zebrafish (*Danio rerio*) embryo has become an essential tool in the study of developmental biology and (eco)toxicology in recent decades. The use of zebrafish embryos as model organisms is justified by their rapid growth outside the mother's body, their transparency, which allows the visualization of morphological changes during development, and their high genetic similarity with several vertebrates (e.g., humans). Ecotoxicology studies using zebrafish embryos as a biological model to analyze developmental, biochemical and behavioral effects usually start exposure in the first hours after fertilization (± up to 3 hours post-fertilization (hpf)) during 96 or 120 h (Abdelkader et al. 2013; Abe et al. 2018; Andrade et al. 2016; Chakraborty et al. 2011; Chollett et al. 2014; De Farias et al. 2021; Godoy et al. 2018; Roberts et al. 2016; Selderslaghs et al. 2012), based on the OECD guideline 236 (OECD 2013). However, some limitations resulting from the presence of the chorion have been described. The chorion can act as a barrier, decreasing the entry of the chemical and consequently the concentration that reaches the embryo, decreasing the toxicity of some contaminants such as pharmaceutical, nanomaterials, and metals/metalloid (Chen et al. 2020; Coral et al. 2021; Halbach et al. 2020; Jeffries et al. 2014; Kämmer et al. 2022; Kristofco et al. 2018; Liegertová et al. 2018; Qiang et al. 2020; Warner et al. 2022). Billat et al. (2023) demonstrated, through physiologically based pharmacokinetic models, the important role of the chorion in the absorption of bisphenol A (BPA) in the first 48 hpf. The model demonstrated that non-chorionated embryos have an increased exposure to BPA, compared to intact embryos. Several studies (e.g., Lammer et al. 2009; Pruvot et al. 2012) have highlighted the importance of considering the developmental stages of the fish, within the limits allowed by European legislation (embryonic stage up to free feeding), in order to avoid problems arising from the different permeability of the chorion to different chemicals or even to reduce the duration of exposure (Embry et al. 2010; Lammer et al. 2009; OECD 2013). The most common approach to address this limitation is by removing the chorion and exposing the "unprotected" embryo. However, embryo dechorionation methods may be aggressive to embryos, and survival rates of dechorionated embryos are typically not subjected to statistical analysis (Henn and Braunbeck 2011). For instance, the mechanical dechorionation process, using fine forceps at 4 hpf (Truong et al. 2022), can be detrimental to embryos and result in a high mortality rate. The use of solutions to digest or dissolve the chorion may also impact the embryos, but there has been limited research in this regard (Henn and Braunbeck 2011). Zebrafish typically hatch between 48 and 72 hpf (Ali et al. 2011; Kimmel et al. 1995). In this sense, the use of zebrafish eleutheroembryos at this developmental stage in toxicity studies offers the advantage of the natural absence of chorion. This makes them particularly valuable in the assessment of the toxicity of substances with limited capacity to penetrate the chorion. Although there are methodological approaches that allow chorion removal (e.g., mechanical removal), there is limited knowledge on its effects on developing embryos. In this sense, with the aim of avoiding the interference of the chorion in the results of toxicological tests, some new methodologies have been proposed. For example, El-Amrani et al. (2012) proposed a protocol to evaluate the bioconcentration of three pesticides, with different chemical and toxicological characteristics (chlorpyrifos, atrazine, and dicofol), in zebrafish eleutheroembryos (from 72 to 120 hpf). Molina-Fernández et al. (2021) developed a miniaturized protocol that used zebrafish eleutheroembryos (from 72 to 120 hpf) to evaluate the bioconcentration, biotransformation of selective serotonin reuptake inhibitors (fluoxetine, sertraline, paroxetine, and citalopram), and their main metabolites (norfluoxetine, norsetraline, and desmethylcitalopram). Massei et al. (2021) investigated the effects of 3 narcosis‐inducing chemicals (phenanthrene, 1,3,5-trichlorobenzene, and pentachlorobenzene) on the respiration of zebrafish eleutheroembryos in a short exposure window (from 58 to 72 hpf) to avoid effects on growth that would influence respiration. According to Lammer et al. (2009), chemicals have the same effect on zebrafish embryos and eleutheroembryos. Some substances, however, such as higher molecular weight polymers and nonionic surfactants, are less predictable, and eleutheroembryos may be more sensitive when exposed to these compounds. On the other hand, Pruvot et al. (2012) demonstrated that embryos in early stages of development (4 hpf) when exposed for 24 h to different chemical products (e.g., theophylline, valproic acid) show lower sensitivity when compared to older embryos (48 hpf). In this context, the best exposure window throughout zebrafish development should be sought, ideally avoiding potential confounding effects generated by exposure during the chorionated phase. Therefore, the hypothesis of this study is that a shorter exposure in the late embryo development period (after hatching) produces effects similar to a longer exposure encompassing the chorionated embryo phase. To test this hypothesis, CAF (1,3,7-trimethylxanthine) was used as test compound. Due to its widespread use, CAF is now considered as an emerging contaminant and an indicator of human activity (Dafouz et al. 2018). In the reported worst case scenarios, the occurrence of CAF in the aquatic system may vary from 0.001 mg.L^−1^ (Nödler et al. 2014) to 0.003 mg.L^−1^ (Nödler et al. 2014) in seawater, from 0.003 mg.L^−1^ (Fang et al. 2019) to 0.005 mg.L^−1^ (Korekar et al. 2020) in estuaries, and from 0.050 mg.L^−1^ (Korekar et al. 2020) to 0.357 mg.L^−1^ (Ferreira 2005) in river water.

Several literature reports account for the effects of CAF in zebrafish embryos at developmental level (delays and anomalies) including hatching delay (Abdelkader et al. 2013; De Farias et al. 2021). Interestingly, a study conducted by Pruvot et al. (2012) exposing 48 hpf embryos for 24 h (from 48 to 72 hpf), found that this CAF exposure in a later stage of development, reduces the size of embryos, causes cardiac deformities, and reduces heart rate, effects similar to those found in embryos exposed to the chemical at early stages of development. Behavioral disruption may be linked with cholinergic disruption. In fact, De Farias et al. (2021) showed that exposure to CAF (from 2 to 168 hpf) inhibits acetylcholinesterase (AChE) activity in zebrafish larvae even at low concentrations (LOEC: 0.009 mg.L^−1^).

Behavior parameters and AChE activity will be used in this work as sensitive parameters to evaluate differences resulting from the exposure scenarios. Furthermore, direct effects of CAF on energy balance have been reported for humans (increased energy expenditure and decreased energy intake) (Harpaz et al. 2017) and for Wistar rats (reduction of the total body fat) (Panchal et al. 2012), but to the authors’ knowledge no data is available for zebrafish. Energy balance, the relation between the energy absorbed and spent by the organism (e.g., in survival, growth and reproduction), allows the assessment of ecological efficiency of a food chain, which starts with the ability of organisms to assimilate food, develop, and reproduce. Therefore, changes in the energetic balance of individuals may be considered an important tool for understanding animal metabolism in stressful situations (Renquist et al. 2013), and provide a warning sign for the ecological community (Baas et al. 2018; Brown et al. 2004).

Thus, this study aimed to assess effects of CAF in different exposure scenarios including early (before hatching) and later (after hatching) exposures. Susceptibility to CAF (0, 13, 20, 44, 67, and 100 mg.L^−1^) considering locomotion behavior and biochemical parameters was compared in three different scenarios: exposure from 2 to 120 hpf (5 days exposure—5dE), exposure from 72 to 120 hpf (2dE), and exposure from 96 to 120 hpf (1dE). In the 5dE scenario, the embryos are surrounded by the chorion when exposure begins, therefore the chorion can operate as a protective barrier during this time (Qiang et al. 2020) decreasing the absorption of CAF by the embryo (Halbach et al. 2020), and within this period the cytochrome P450 activity (CYP1; involved in the oxidative metabolism (phase I) of xenobiotics) is detected (approximately between 7 and 26 hpf) (Verbueken et al. 2018). The 2dE scenario (from 72 to 120 hpf) is a post-hatching exposure period that coincides with the opening of the mouth, a potential additional portal of entry for CAF. The 1dE scenario (exposure from 96 to 120 hpf) is a later exposure period when other CYP (*CYP2ad2, 2k18, 2n13, 3a65, 3c2/3*, and *3c4*) transcripts increase suddenly, and a period closest to the total formation of the liver (i.e., 120 hpf)(Nawaji et al. 2020; Verbueken et al. 2018), and simultaneously present potential new drug targets (Pruvot et al. 2012). To assess the reversibility of potential CAF effects, after each exposure period, some fish were subjected to a 10 days depuration period in clean water (10dpE)—the predicted time for complete elimination of CAF in human newborns (Christian and Brent 2001). The endpoints evaluated were behavioral parameters (total distance moved, swimming pattern (distance moved at the edges of the well), and erratic swimming), and acetylcholinesterase (AChE) activity at the end of the exposure and depuration periods. Moreover, to evaluate the impacts of CAF on the general health of the fish, the energy balance was also evaluated in scenario 5dE.

## Material and methods

### Test organisms

Zebrafish (*D. rerio*) eggs were obtained by natural mating of adult fish (wildtype AB; 5-month-old fish) from a culture established at the Biology Department of University of Aveiro (Portugal). Adult fish are kept in a recirculation system, with a photoperiod cycle of 12:12 h (light:dark), water temperature of 27.0 ± 1 °C, conductivity of 750 ± 50 μS/cm, pH 7.0 ± 0.5 and dissolved oxygen saturation higher than 95%. Adult fish were fed once a day with a commercial diet Gemma Micro 500 (Skretting®, Spain). Zebrafish eggs were collected within 30 min after mating, washed in fish system water and screened with the help of a stereomicroscope (Stereoscopic Zoom Microscope-SMZ 1500, Nikon Corporation) to eliminate unfertilized and damaged embryos. In this sense, eggs having cleavage defects, lesions, or other sorts of abnormalities were eliminated.

### Chemicals

Caffeine (CAF) (1,3,7-Trimethylxanthine; CAS number 58–08-2) used was of analytical grade (98% purity) and acquired from TCI Chemicals (Belgium). The stock solution (1000 mg.L^−1^) was prepared in zebrafish water system (see the Sect. 2.1). Test solutions were prepared by successive dilutions of the stock solution in culture water.

### Experimental design

Three exposure scenarios were tested following procedures based on the OECD guideline 236 on Fish Embryo Toxicity Test (FET) (OECD 2013): i) exposure from 2 to 120 h post-fertilization (hpf)—5 days of exposure (5dE), ii) exposure from 72 to 120 hpf—2 days of exposure (2dE), and iii) exposure from 96 to 120 hpf—1 day of exposure (1dE). Based on a preliminary assay that considered only the scenario described in OECD guideline 236 (OECD 2013) no behavioral changes were found in organisms exposed to CAF concentrations equal or below to 10 mg.L^−1^ (data not shown), the following concentrations of CAF were tested: 0, 13, 20, 30, 44, 67, and 100 mg.L^−1^ of CAF. To evaluate behavior, in each scenario (5 days (5dE), 2 days (2dE) and 1 day (1dE)), 24 fish per concentration were exposed to CAF in 24-well plates, containing 2 mL of the test solution. To evaluate acetylcholinesterase (AChE) activity, a total of 150 fish per concentration were used in each scenario. These fish were exposed to CAF in Petri dishes, with three replicates per concentration (50 embryos in 25 mL of solution). In the analysis of energy reserves in the 5dE scenario, 300 fish per concentration were used, and the fish were exposed to CAF in Petri dishes with six replicates per concentration (50 embryos in 25 mL of solution). The same physicochemical conditions were maintained for both the behavioral and biochemical tests. Water temperature was 27 ± 1 ºC, pH at 7.5 ± 0.5, dissolved oxygen equal or above 95% saturation, a photoperiod of 12:12 (light:dark), and a conductivity of 750 ± 50 μS. CAF has a half-life in a microcosm composed of fish, aquatic plants, zooplankton, phytoplankton, macrophytes and bacteria of 1.7 ± 0.2 days, in this sense, the medium was renewed every 48 h to maintain constant CAF concentrations (Lam et al. 2004). In human newborns, the half-life of CAF is 4 days, while it takes 10 days for all ingested CAF to be removed from the body (Christian and Brent 2001). Based on this information a 10-days depuration period was selected in this study. At the end of each assay, in the 3 exposure scenarios, half of the larvae were transferred to clean medium (culture water, see the Sect. 2.1) for a recovery assessment after 10 days (10dPE) and the other half were immediately used for endpoints assessment. Only larvae with no malformations were used for behavioral assays.

### Heartbeat count

In the 5dE scenario, the heartbeat of 10 embryos per treatment was analyzed at 48 hpf using a stereomicroscope (Stereoscopic Zoom Microscope-SMZ 1500, Nikon Corporation). Heartbeats were measured by counting heartbeats over a period of 15 s (n = 10), according to Zhu et al. (2007).

### Behavior analysis

It is known that zebrafish activity is influenced by plate circumference. For example, previous research has reported that zebrafish exhibit greater swimming activity in 24-well plates compared to other plate sizes such as 48 or 12 wells (Lovin et al. 2023). This variability in swimming behaviour could potentially result in differences in anxiety and thigmotaxis responses between studies. In this regard, the present study opted to use 24-well plates, as they are recommended by OECD 236 and have established protocols for analysing thigmotactic behaviour (OECD 2013; Schnörr et al. 2012) . Assays for behavior analysis were deployed with 2 mL of the test solution per well, one embryo per well (48 embryos were used per concentration) and under controlled conditions of temperature (27 ± 1 °C) and photoperiod (12:12 h light: dark).

Behavior was analyzed after the exposure period and after the recovery period in each scenario using the Zebrabox video tracking system (software version 3.22, Viewpoint Life Sciences, Lyon, France), which is equipped with a 25-images-per-second infrared camera. Generally, zebrafish larvae have low levels of activity under light conditions and react with a burst of activity upon a sudden switch to darkness. The 24-wells plates were placed in the equipment and the movement was recorded during 3 min in the dark, after a 3 min acclimatization period in the light, following the protocol described by Correia et al. (2019).

To evaluate the swimming pattern analysis, swimming activity was studied in two distinct areas of each well: an inner circle (± 12.2 mm of ray) and an outer ring (± 4 mm). The tendency to swim near the edges of the well (as a measure of thigmotaxis), was calculated dividing the distance moved in the outer zone by the total swimming distance and multiplying by 100 (Schnörr et al. 2012).

**Fig. 1:**
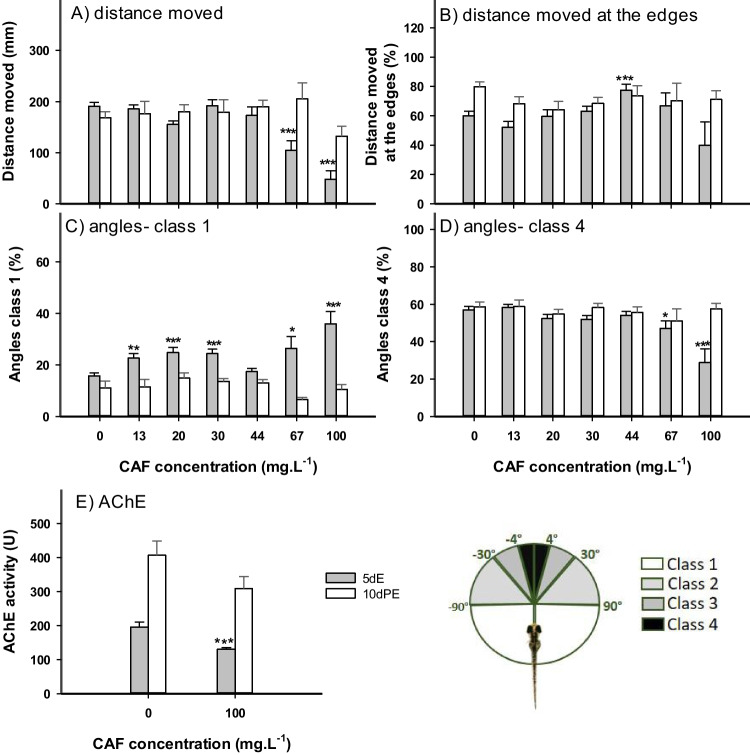
Effects of caffeine (CAF) on zebrafish eleutheroembryos after 5 days exposure—from 2 to 120 hpf—(5dE) (grey bars), and 10 days depuration period (10dPE- white bars). A) Total distance moved by larvae of zebrafish B) Percentage of distance moved by larvae near the edges of the well (n = 24). C) Percentage of class 1 angles (n = 24). D) Percentage of class 4 angles (n = 24). E) Acetylcholinesterase (AChE) activity (n = 10). F) Scheme of the angle’s classes. Results are expressed as mean values ± standard error. Asterisks (*) represent significant differences to the control (*—*p* < 0.05, **—*p* < 0.01, ***—*p* < 0.001)

The fish path angles were analyzed according to Almeida et al. (2019) using 4 classes of angles: class 1 that includes angles from 180º to 90º and -180º to -90º, corresponding to movement with abrupt changes of direction or erratic swimming; class 2 corresponding to angles from 90º to 30º and -90º to -30º; class 3 that includes angles from 30º to 4º and -30º to -4º; and class 4, with angles from 4º to 0º and 0º to -4º representing movements without abrupt changes of direction (Fig. 1F).

### Biochemical analysis

For AChE determination, 3 Petri dishes (each containing 50 embryos and 25 mL of test solution) per treatment (0 and 100 mg.L^−1^ of CAF) were used in each scenario (5dE, 2dE, and 1dE). AChE was analyzed (n = 10) after the exposure, and after the depuration period. Each replicate consisted in a pool of 5 larvae which were transferred to 2 mL microtubes, immediately frozen in liquid nitrogen, and stored at -80 ºC until further processing. AChE was only analyzed at 100 mg.L^−1^ since it is a concentrations known to inhibit this enzyme (literature reports inhibition of AChE activity in zebrafish both at low (0.009 mg.L^−1^) and high concentrations (291.3 mg.L^−1^) (De Farias et al. 2021; Teixidó et al. 2013). The degree of enzymatic inhibition among the different scenarios and control group was then calculated.

AChE samples were thawed on ice, homogenized in 500 µL of phosphate buffer (0.2 M, pH 7.4) with a sonicator (KIKA Labortechnik U2005 Control) and centrifuged at 4 °C, 10,000 *g*, 20 min). The supernatant was used to determine AChE activity at 414 nm as described by Domingues et al. (2010), based on the method of Ellman et al. (1961). AChE activity was normalized by the sample protein content, quantified using the Bradford method (Bradford 1976). Activity was expressed as unit (U), with a U corresponding to a nanomol of substrate hydrolyzed per min per milligram of protein.

For energy reserves determination, 6 Petri dishes (each containing 50 embryos and 25 mL of test solution) per treatment (0, 13, 20, 30, 44, 67, and 100 mg.L^−1^ of CAF) were used in the 5dE scenario. Energy reserves were analyzed (n = 10) only after the exposure. Each replicate consisted in a pool of 15 larvae which were transferred to 2 mL microtubes, immediately frozen in liquid nitrogen, and stored at -80 ºC until analysis for further processing. Energy reserves in organisms exposed in the 5dE scenario were studied, in order to characterize the general health status of the fish, as previous studies describing the effects of CAF on energy reserves in embryos were not found.

Energy reserves (Ea) samples were thawed on ice, homogenized in 1000 µL of ultra-pure water with a sonicator (KIKA Labortechnik U2005 Control). The energy budget was determined through the sum of lipids, carbohydrates, and proteins, converted into energetic equivalents using the correspondent enthalpy of combustion (39.5 kJ/g lipids, 17.5 kJ/g glycogen and 24 kJ/g for proteins). Total lipids were measured at 375 nm using tripalmitin as standard; total carbohydrates were quantified at 492 nm adopting glucose as standard, and protein content was determined at 592 nm using bovine serum albumin as standard, as described by Rodrigues et al. (2015) based on the methods of De Coen and Janssen (1997). The energy reserves were expressed in mJ/ organism.

Energy consumption (Ec) was analyzed based on electron transport system (ETS), according to the method described by De Coen and Janssen (1997). ETS was kinetically determined at 490 nm for 3 min (De Coen and Janssen 1997). The activity was expressed in mJ/org/hour.

Cellular energy allocation (CEA) was calculated according to Verslycke et al. (2003) using the formula: CEA = Ea/Ec and the results were expressed in mJ / organism.

To evaluate the Ea, a larger pool of larvae (15 larvae) was necessary, as the samples were divided into aliquots (300 µL) to determine the Ea parameters (lipids, carbohydrates, and proteins) and the Ec.

All biochemical determinations were done using spectrophotometric methodologies adapted to 96 well microplates and using a Thermo Scientific Multiskan Spectrum (USA).

### Statistical Analysis

Data was analyzed using Sigma Plot V.12.5 statistical package (SysStat, San Jose, California, USA). A one-way analysis of variance (ANOVA) was performed to investigate the effects of CAF on the parameters evaluated in normally distributed data sets. If significant effects were found, the Dunnett test was used to verify differences between treatments and control. Whenever the data did not pass the normality test, the Kruskal–Wallis test was performed, followed by Dunn's multiple comparison to assess differences towards control. Results of the statistical tests can be seen in Table S1.

## Results

Survival rate, hatching, and development were not significantly affected by exposure to CAF.

### Effects of CAF after 5dE

After 5 days exposure to CAF, the distance moved by the organisms exposed to 67, and 100 mg.L^−1^ was significantly lower than controls, corresponding respectively to a 45.2, and 74.7% decrease (Fig. 1A). The larvae exposed to the highest concentration tested (100 mg.L^−1^) were mainly still, displaying constant spasms, a symptom noticeable shortly after hatching until the end of the test. After the depuration period (10dPE), a significant recovery in the locomotion was observed, with the distance moved by organisms exposed to 67, and 100 mg.L^−1^, returning to levels close to the controls (Fig. 1A).

Regarding spatial distribution (Fig. 1B), larvae from the intermediary concentrations (20, 30, 44, and 67 mg.L^−1^) showed a trend to increase movement at the edges of the wells, although only significantly in organisms exposed to 44 mg.L^−1^. This behavior was not observed after the depuration period.

In terms of swimming angles (Fig. 1C), the larvae exposed to CAF presented, when compared to controls, higher percentages of class 1 angles (13, 20, 30, 44, 67, and 100 mg.L^.−1^), and lower percentage of class 4 angles (67, and 100 mg.L^−1^) (Fig. 1D). No significant effects in terms of class 2, and 3 were found after exposure to CAF. After the 10dPE, no significant effects of CAF in terms of swimming angles remained noticeable.

Larvae from 5dE scenario, exposed to 100 mg.L^−1^, had significantly lower AChE activity, when compared to control (33.6% decrease). AChE activity in controls increased from 195 U in 5 days old larvae to ~ 400 U in 15 days old larvae. The depuration period allowed the recovery of activity to levels similar to controls (Fig. 1E).

**Fig. 2:**
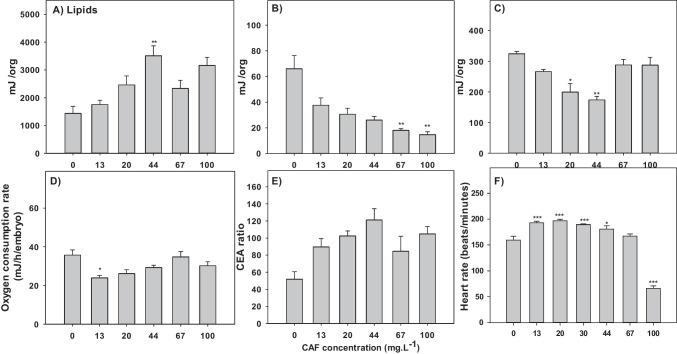
Effects of caffeine (CAF) exposure on the energy reserves (n = 10) and heartbeat of zebrafish (n = 10). A) Lipids content. B) Carbohydrates content. C) Proteins content. D) Electron transportation system (ETs). E) Cellular energy allocation (CEA). F) Heartbeat. Results are expressed as mean values ± standard error. Asterisks (*) represent significant differences to the control (*—*p* < 0.05, **—*p* < 0.01, ***—*p* < 0.001)

The effects of zebrafish 5dE to CAF on energy reserves is depicted in Fig. 2. Exposure to CAF resulted in a trend to increase the content of lipids, although only significantly for 44 mg.L^−1^ CAF exposed organisms (Fig. 2A). An opposite trend was found in terms of carbohydrates content, which significantly decreased in 67, and 100 mg.L^−1^ exposed organisms (Fig. 2B). The proteins content showed an irregular response with a significant decrease at intermediary concentrations (20, and 44 mg.L^−1^) (Fig. 2C). The ETS was significantly inhibited in larvae exposed to 13 mg.L^−1^ CAF (Fig. 2D). No significant differences were observed in CEA (Fig. 2E). A significant increase in heart rate was observed in the organisms exposed to 13, 20, 30, and 44 mg.L^−1^ (Fig. 2F). However, embryos exposed to 100 mg.L^−1^ CAF showed a significant decrease in the heart rate. In these embryos (100 mg.L^−1^), oedema of the pericardium was observed at 72 hpf, an effect not observed at 120 hpf (data not shown).

### Effects of CAF after 2dE

**Fig. 3:**
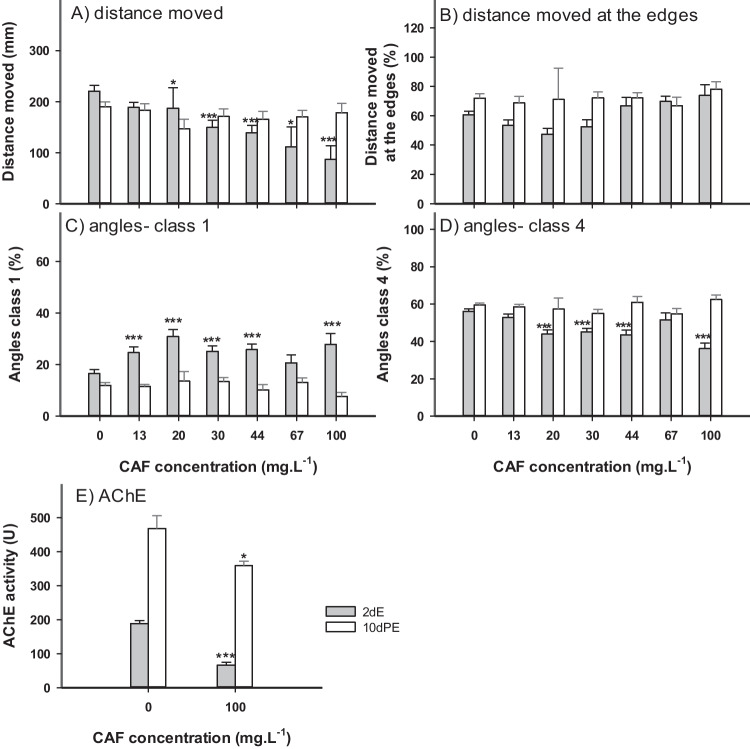
Effects of caffeine (CAF) on zebrafish eleutheroembryos exposed from 72 to 120 hpf (2 days exposure- 2dE) (grey bars), and 10 days post-exposure (10dPE- white bars). A) Total distance moved by larvae of zebrafish (n = 24). B) Percentage of distance moved by larvae near the edges of the well (n = 24). C) Percentage of class 1 angles (n = 24). D) Percentage of class 4 angles (n = 24). E) Acetylcholinesterase (AChE) activity (n = 10). Results are expressed as mean values ± standard error. Asterisks (*) represent significant differences to the control (*—*p* < 0.05, **—*p* < 0.01, ***—*p* < 0.001)

Upon exposure to CAF, from 72 to 120 hpf (scenario 2dE) larvae showed a significant reduction in the total distance moved (20, 30, 44, 67, and 100 mg.L^−1^) (Fig. 3A) reaching, when compared to control organisms, maximum inhibition of 60.6% in the organisms exposed to 100 mg.L^−1^. However, after 10dPE no differences to control group were found. No significant effects of CAF on the swimming pattern (center vs edges of the well) were observed, although a trend to increase distance in the outer zone was observed in the organisms exposed to concentrations higher than 44 mg.L^−1^ after 2 dE (Fig. 3B). As observed in the 5dE scenario, after CAF exposure, class 1 angles were significantly increased at all concentrations (13, 20, 30, 44, 67, and 100 mg.L^−1^) (Fig. 3C) whereas class 4 were significantly decreased in organisms exposed to 20, 30, 44, and 100 mg.L^−1^ (Fig. 3D). Class 2, and 3 angles showed no significant alterations when compared to control (data not shown). The effects observed in classes 1, and 4 were not persistent after the depuration period (10dPE).

In the 2dE scenario, AChE activity was significantly inhibited (64.7%) in larvae exposed to 100 mg.L^−1^. After the depuration period a lower enzymatic activity was still observed, although less intense (only of 23.3%) (Fig. 3E).

### Effects of CAF after 1dE

**Fig. 4:**
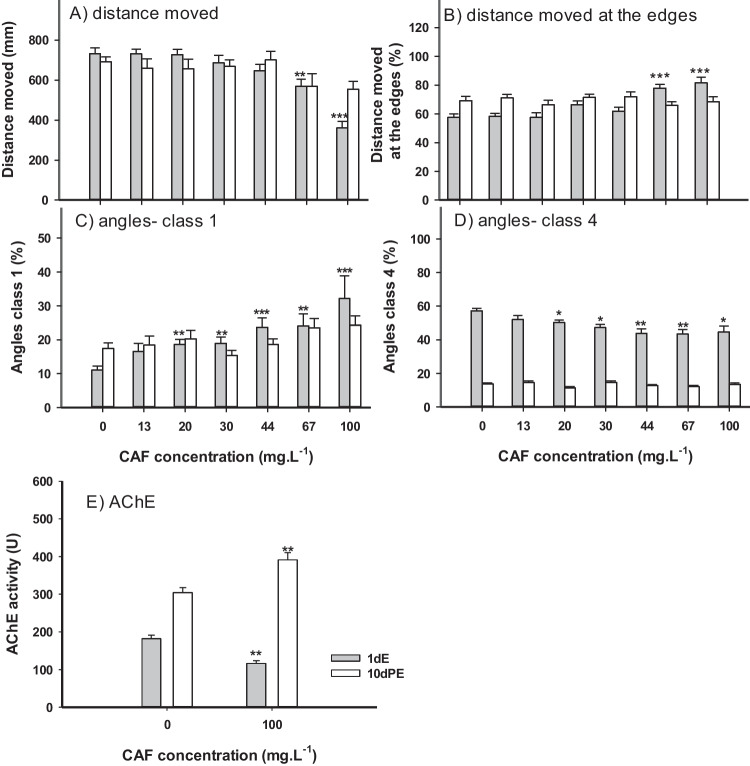
Effects of caffeine (CAF) on zebrafish eleutheroembryos from 96 to 120 hpf (1 day of exposure- 1dE) (grey bars), and 10 days post-exposure (10dPE- white bars). A) Total distance moved by larvae of zebrafish (n = 24). B) Percentage of distance moved by larvae near the edges of the well (n = 24). C) Percentage of class 1 angles (n = 24). D) Percentage of class 4 angles (n = 24). E) Acetylcholinesterase (AChE) activity (n = 10). Results are expressed as mean values ± standard error. Asterisks (*) represent significant differences to the control (*—*p* < 0.05, **—*p* < 0.01, ***—*p* < 0.001)

Exposure to CAF, from 96 to 120 hpf, showed a general tendency to affect the locomotor behavior of zebrafish larvae. A reduction of the total distance moved by larvae was observed after exposure to 67, and 100 mg.L^−1^ (24%, and 51% reduction of movement compared to control respectively (Fig. 4A)). The depuration period (10dPE) led to a full recovery of the effects observed in this parameter. In this exposure scenario, larvae increased the movement near the edges of the well (increased thigmotaxis) after exposure to 67, and 100 mg.L^−1^ (33%, and 41% movement increase when compared to control, respectively) (Fig. 4B). This increment was no longer observed after the depuration period (10dPE).

In 1dE exposure scenario organisms exposed to concentrations higher than 13 mg.L^−1^ showed higher percentages of class 1 angles, and lower percentages of class 4 angles. However, these effects were no longer observed after 10dPE period (Fig. 4C and D). For classes 2, and 3 no significant effects were observed (data not shown).

Concerning AChE activity, CAF induced a significant inhibition of AChE activity (36.3% lower than control) after 1dE of exposure to 100 mg.L^−1^ CAF. However, a significant enzymatic activity increase (28.8% higher than control) was observed after 10dPE (Fig. 4E).

## Discussion

This study aimed to assess the toxicity of CAF to embryos exposed in a conventional scenario (2–120 hours post fertilization (hpf)) (5 days of exposure- 5dE) encompassing the period inside the chorion as opposed to embryos already hatched (72–120 hpf (2dE), and 96–120 hpf (1dE)); and the reversal of potential effects after a 10 days depuration period (10dPE).

CAF is a central nervous system stimulant. Its main mode of interaction is antagonism with adenosine receptors (A_1_, A_2_, A_3_, and A_4_) (El Yacoubi et al. 2000). This interaction can be either excitatory when affecting A_1_ receptors, or inhibitory when acting on A_2_ receptors (Rita Costenla et al. 2010). Additionally, CAF acts as a non-competitive inhibitor of AChE and influences calcium homeostasis (Daly 2007; Pohanka 2014; Pohanka and Dobes 2013). However, CAF also interacts with other metabolic pathways, such as gamma-aminobutyric acid (GABA) receptors (Daly 2007).

Exposure to CAF led to a decrease in the total distance moved by larvae in all the scenarios tested (5dE, 2dE, and 1dE), which suggests hypoactivity, a commonly described effect of CAF for both larvae and adult zebrafish (Cruz et al. 2017; Rosa et al. 2018; Tran et al. 2017). However, hypoactivity in the 2dE scenario was observed at lower concentrations (lowest observed effect concentration (LOEC): 20 mg.L^−1^) while in the 5dE, and 1dE scenarios it occurs exclusively at the highest concentrations evaluated (67, and 100 mg.L^−1^), suggesting higher sensitivity of the eleutheroembryos in the 2dE scenario. On the other hand, the inhibition of the movement was, at the highest concentration tested (100 mg.L^−1^), when compared to the respective controls, approximately 75, 60, and 51% in 5dE, 2dE, and 1dE scenarios respectively, suggesting that the degree of inhibition or the intensity of inhibition in organisms exposed to high concentrations (100 mg.L^−1^) is proportional to the exposure duration. The presence of chorion has been considered as a protective barrier that provides security to the embryo limiting the entrance of chemicals (Halbach et al. 2020; Qiang et al. 2020). In this sense, it has been described that xenobiotic presence in zebrafish embryos is low before 72 hpf (Verbueken et al. 2018). For instance, Chen et al. (2008) reported that in zebrafish embryos exposed by immersion to 150 mg.L^−1^ of CAF for 2 days, only 172.5 ng of CAF entered the embryo. Moreover, the results obtained in the present study (both intensity and sensitivity) may also be associated with the rate of CAF metabolism by the eleutheroembryo. In humans, CAF is metabolized and methylated in the liver microsomes by cytochrome P450 (CYP1A2 isoform)(Gu et al. 1992; Magkos and Kavouras 2005; Nehlig 2018). In zebrafish, the metabolism of CAF in the early stages of embryonic development occurs slowly and depends on the expression of CYPs (Nawaji et al. 2020). For example, in zebrafish exposed (from 0 to 120 hpf) to 10 mg.L^−1^ the conversion of CAF to 1,7-dimethylxanthine was detected at 50 hpf (Nawaji et al. 2020). On the other hand, significant CYP activity has been detected between 7 and 26 hpf (*CYP1*), while increased expression of other CYPs *(CYP2ad2, 2k18, 2n13, 3a65, 3c2/3,* and *3c4*) has been described around 96 hpf (Nawaji et al. 2020; Verbueken et al. 2018). This suggests that CAF may be biotransformed even before 50 hpf. In this regard, it is suggested that the presence of the chorion, which limits CAF penetration into the embryo during the early 72 hpf, combined with the metabolization of CAF by CYPs, may contribute to the reduction of CAF toxicity in lower CAF concentrations (13, 20, 30, and 40 mg.L^−1^) in the 5dE scenario. However, in the 5dE scenario embryos exposed to higher concentrations of CAF (67, and 100 mg.L^−1^), a significant amount of compound may manage to reach embryos, which due to longer exposure duration and limited metabolization capacity, may end up accumulating CAF in the body and therefore display more effects than to eleutheroembryos subjected to the same concentrations in the 1dE, and 2dE scenarios. CAF bioaccumulation was detected when zebrafish embryos were dechoronated and exposed to 10 mg.L^−1^ of CAF for approximately 120 h (Nawaji et al. 2020). Conversely, the eleutheroembryos from the 2dE scenario, much like their counterparts in the 5dE scenario, exhibit a low metabolic rate at the start of exposure due to the fact that the increase in fish metabolization rate in the 2dE scenario appears to begin at 74 hpf but becomes more pronounced only at 96 hpf when there is a significant increase in the expression of new CYPs (Nawaji et al. 2020; Verbueken et al. 2018). Nevertheless, in contrast to the 5dE scenario, fish in 2dE were exposed to CAF through multiple routes because, in addition to dermal exposure, their mouths were open during the exposure period. Thus, in this scenario (2dE), similar to what occurred with organisms exposed to high concentrations of CAF in the 5dE scenario, CAF accumulation would occur even in organisms exposed to low concentrations (20 mg.L^−1^), which would explain the effects of CAF on behavior of organisms of this scenario (2dE). In the 1dE scenario, basal biotransformation ability associated with CYP1 and other CYPs (*cyp2ad2, 2k18, 2n13, 3a65, 3c2/3* and *3c4*) may endow organisms with a higher protection than 2dE (Nawaji et al. 2020; Verbueken et al. 2018). In this regard, eleutheroembryos from the 1dE scenario, at the beginning of the exposure may be more capable of CAF than organisms from the 2dE scenario, due to a significant increase in CYPs expression at 96 hpf or by the more advanced formation of liver and intestine- organs involved in xenobiotic biotransformation (James et al. 2005; Nawaji et al. 2020; Verbueken et al. 2018; Wallace et al. 2005). Alternatively, the greater sensitivity of eleutheroembryos in the 2dE scenario, when compared to those in the 1dE scenario, may result from exposure at an earlier stage of development and/or even more prolonged exposure. Pruvot et al. (2012) discovered that zebrafish eleutheroembryos exposed to CAF during the early stages of development (from 4 to 24, and from 4 to 48 hpf) display sensitivity equivalent to that reported in eleutheroembryos exposed to CAF at later stages of development (from 48 to 72 hpf). Our results differ from the results found by Pruvot et al. (2012), however, these inconsistencies may result from differences in exposure scenarios of the theses authors (from 4 to 24 hpf; from 4 to 48 hpf; and 48 to 72 hpf) and those evaluated in the present study (from 2 to 120 hpf; from 72 to 120 hpf; and from 96 to 120 hpf). On the other hand, at the behavioral level Pruvot et al. (2012) only analyzed the locomotor activity of eleutheroembryos 4 days after exposing the organisms to CAF, which could have influenced the results found.

Increased thigmotaxis behavior is indicative of anxiety in animals (Schnörr et al. 2012). The swimming pattern of the larvae in what concerns the spatial distribution in the wells (i.e., center vs edges) was not a consistent parameter across the different exposure scenarios, although an overall increment of thigmotaxis after exposure to CAF was more noticeable in the 1dE scenario. In general, thigmotaxis, seems to be a parameter of anxiety most easily detected in the first hours of fish exposure to CAF (Schnörr et al. 2012; Richendrfer et al. 2012; Rosa et al. 2018), which agrees with the 1dE scenario results. On the other hand, the increase in the percentage of class 1 angles (indicating a zigzag swimming pattern) along with a reduction in the percentage of class 4 angles (which represents movements without abrupt changes of direction) also suggest an increase in anxiety in the fish (Almeida et al. 2019). Furthermore, change in the swimming pattern (class of angles) may also be indicative of neuronal disruption, such as defects in axonal growth of the primary motor neuron and secondary motor neuron axon projection defects (Chen et al. 2008) or representative of a muscle fiber disorder (Chen et al. 2008). In this study, changes in swimming angles were detected in all scenarios (5dE, 2dE, and 1dE) suggesting that CAF induces erratic movements in zebrafish in different scenarios. However, effects in the locomotion angles were more prominent in the 2dE, and 1dE scenarios, characterized by an increase in high-amplitude angles (class 1), as well as a reduction in low-amplitude angles (class 4) at all CAF concentrations. Such results (alterations in class 1 and 4 angles) can indicate the presence of anxiety or neuronal disruption after exposure of eleutheroembyos to all CAF concentration in shorter exposure scenarios. On the other hand, in the 5dE scenario, only eleutheroembryos exposed to high concentrations of CAF (67, and 100 mg.L^−1^) followed a pattern of response similar to organisms in the 2dE and 1dE scenario (increased class 1 angles, and decreased class 4 angles), which is consistent with the reduction in the total distance moved, and can also result from the accumulation of CAF in these organisms during exposure. In general, in this study, a decrease in the total distance moved, and changes in swimming angles, in organisms exposed to the highest concentration of CAF (100 mg.L^−1^) occurred concomitantly with the inhibition of AChE activity in all scenarios. The inhibition of AChE activity reduces the cleavage of the acetylcholine into choline and acetate, and consequently increases acetylcholine in the synaptic cleft (Thapa et al. 2017), leading to symptoms such as paralysis that can progress to convulsions or cardiac and respiratory depression, potentially resulting in death (Tilton et al. 2011). Eleutheroembryos exposed to CAF (100 mg.L^−1^) in all scenarios also presented constant spasms, responses characteristic of the action of acetylcholine on nicotinic receptor that opens ionic channels and induces muscle contraction. In contrast to the distance moved, a greater inhibition of enzymatic activity was detected in embryos from the 2dE scenario AChE (5dE: 33.6%, 2dE: 64.7%, 1dE: 36.6%). As the biochemical response (AChE) occurs before an individual-level response, greater AChE inhibition would be predicted in eleutheroembryos from later stages (2dE and 1dE), since they were exposed to CAF late and in a period closer to 120 hpf, when the test would be completed (Weis et al. 2001). The greater inhibition, detected simultaneously with behavioral effects, in the 2dE scenario, may be associated with a delay in CAF metabolization by these eleutheroembryos, as discussed in the previous section. In agreement with our results, an increase in erratic movements was observed in zebrafish eleutheroembryos exposed for approximately 120 h (between 2 and 122 hpf) and 168 h (between 2 and 170 hpf) to 2.8, and 50 mg.L^−1^ of CAF (De Farias et al. 2021). Chen et al. (2008), studied the effects of CAF (17.5, 35, 150 mg.L^−1^) on muscle fibers in eleutheroembryos in different exposure scenarios (0–24; 0–36; 0–48; 24–48 hpf). In the longest exposure scenario (0–48 hpf) alterations were detected in muscle fibers of eleutheroembryos exposed to 150 mg.L^−1^ of CAF, while in the scenario where the eleutheroembryos were exposed from 24 to 48 hpf, these changes were detected at low concentrations (17.5, and 35 mg.L^−1^). Similar to our results on angles, and despite different exposure periods, the developmental stage in which the eleutheroembryos were exposed to CAF seems to influence its effects on muscle fibres, suggesting that the chorion and metabolism of CAF reduce CAF toxicity in embryos in the early stages of development. Probably, the presence of chorion in the 5dE and CAF metabolizing capacity in the 5dE and 1dE scenarios works as a similar protection mechanism in eleutheroembryos in the two scenarios, as both present similar results (distance moved; distance out %, AChE activity), when compared to the eleutheroembryos in the 2dE scenario.

In order to understand the general state of health of the fish, we evaluated the heart rate and energy reserves. In this study, a biphasic effect of CAF was also detected with the increase of heart rates at low CAF concentrations (13, 20, and 30 mg.L^−1^) and a decrease at the highest concentration (100 mg.L^−1^). In line with our findings, Maeda et al. (2021) also observed a biphasic effect of CAF on heart rate. Lower concentrations of CAF (0.1, and 1 mg.L^−1^) increased heart rate, while higher concentrations (300, 500, and 1000 mg.L^−1^) reduced it. The authors suggested that CAF-induced bradycardia may occur through cholinergic activation by AChE inhibition or ether-a-go-go potassium channel inhibition, impacting cardiac repolarization, and potentially causing bradycardia and cardiac arrest. However, the authors also relate the inhibition of the ether-a-go-go potassium channel to the presence of tachycardia and sudden death. Chakraborty et al. (2011) observed that in zebrafish eleutheroembryos, an increase in heart rate induced by CAF (10, 20, and 50 mg.L^−1^) at 72 hpf is associated with a decrease in the expression of vascular endothelial growth factor- a protein involved in the development of heart blood vessels and endothelial muscle development, which results in abnormalities in the heart or in the development of cardiac blood vessels and, consequently, increased heart rate. Contrary to our findings, Subendran et al. (2021) observed a decrease in the heart rate of zebrafish larvae at 5, 7, and 9 dpf when exposed, for 10 min, to 25 mg.L^−1^ of CAF. The authors suggest that CAF effects on zebrafish heart rate are due to the release of calcium in the cytoplasm, which could lead to cardiac nerve transmission and, consequently, a reduction in heart rate. On the other hand, eleutheroembryos of zebrafish exposed to CAF (from 48 to 72 hpf) (LOEC: 582.6 mg.L^−1^) and theophylline—CAF metabolite (from 48 to 72 hpf) (LOEC: 901 mg.L^−1^), have a reduction in heart rate (Pruvot et al. 2012). The action of acetylcholine on muscarinic receptors present in cardiac muscle cells occurs as an indirect action that inhibits the postsynaptic cell, causing hyperpolarization, and consequently a decrease in heart rate (Stengel et al. 2000). In this regard, the decrease in heart rate, together with the neuronal disruption verified by AChE inhibition, suggest that the cholinergic system plays a role in the mechanism of CAF toxicity.

CAF influences the energy balance, as it increases the energy spent and decreases energy intake (Harpaz et al. 2017). Our results demonstrated that eleutheroembryos exposed to CAF (67, and 100 mg.L^−1^) presented lower carbohydrates, suggesting that glucose is used as source of energy to face the stress posed by CAF exposure and increased energy expenditure. This increased energy expenditure may result from the increased muscle contraction detected in these animals. Lipids appear to be the last source of energy consumed by zebrafish after exposure to CAF, since under normal conditions, lipids (via lipolysis) provide energy, and cellular components for the development of vertebrates (Fraher et al. 2016). Additionally, a downward trend was detected in the electron transport system (ETS). A study performed by He et al. (2020) showed that pharmaceuticals (e.g., propofol) induced neurotoxicity in zebrafish embryos can occur by inhibition of the electron transport chain. An inhibition in the electron chain promotes mitochondrial dysfunction, which results in decreased energy production.

To assess the zebrafish's ability to recover from CAF effects, the larvae were maintained for 10 days in culture water (10dPE). The behavioral changes observed in this study were reversed in all scenarios. AChE activity returned to baseline levels only in the 5dE scenario. Recovery of the larvae, in the 5dE scenario (AChE, and Behavior), contributes to the hypothesis that the CAF in these eleutheroembryos has its metabolism initiated at 50 hpf (Nawaji et al. 2020; Verbueken et al. 2018), which suggests a reduction in CAF available earlier when compared to eleutheroembryos of the other two scenarios (2dE and 1dE). A significant increase in AChE activity in the larvae of the 1dE scenario after the depuration period (10dPE) was detected, characteristic of a compensatory mechanism responsible for decreasing the availability of acetylcholine at the neuromuscular junction (Erb et al. 2001). On the other hand, larvae of scenario 2dE at the beginning of exposure (72 hpf), could be considered more susceptible, due to the low biotransformation of CAF (low expression of CYPs and biotransformation organs starting to grow (Kimmel et al. 1995; Verbueken et al. 2018; Wallace et al. 2005) which could suggest a greater accumulation of the CAF inside the fish, and consequently longer-lasting CAF effects.

Therefore, we suggest that late-stage eleutheroembryos (2dE, and 1dE) can be included in toxicity tests, as they are more sensitive to the presence of the chemical in the water compared to early-stage embryos (5dE). Nevertheless, a more specific examination, such as molecular analysis, is required to demonstrate the higher sensitivity of these scenarios.

## Conclusion

This study evaluated whether different developmental stages (exposure onset at 2, 72, and 96 hours post fertilization (hpf) and end at 120 hpf) of zebrafish embryos/eleutheroembryos have distinct susceptibility to CAF. In general, eleutheroembryos showed similar patterns of behavioral changes after exposure to CAF: decrease of total distance moved in all scenarios (5dE, 2dE, and 1dE) and increased erratic movements. Despite the similarity in the pattern of responses to CAF, eleutheroembryos from the 5dE scenario were less sensitive to CAF than organisms from the other two scenarios (2dE, and 1dE). The highest sensitivity to CAF was detected in eleutheroembryos in the 2dE scenario, with the smallest LOEC (20 mg.L^−1^) found in the total distance moved and greater inhibition of AChE activity (65%). Some similarity between the 5dE and 1dE scenarios has been detected in AChE activity and distance moved, likely as a result of lower CAF availability in these organisms. On the other hand, eleutheroembryos at advanced stages of development (2dE, and 1dE) respond to the presence of CAF even at low concentrations. Therefore, considering that a later exposure to CAF rendered the same sensitivity as the traditional exposure scenario, the adoption of this exposure window in toxicity studies may be advantageous in the study of the effects of other chemicals. Nonetheless, additional studies should be performed to confirm this response pattern in chemicals with different modes of action and metabolization rates.

### Supplementary Information

Below is the link to the electronic supplementary material.

Supplementary file1 (DOCX 48 KB)

## Data Availability

The data sets used in the current study are available from the corresponding author upon request.
