# Supplementary material for: Influence of exposure scenario on the sensitivity to caffeine
Source: Environ Sci Pollut Res Int. 2023 Nov 17;30(58):122808–21. doi: 10.1007/s11356-023-30945-3 (PMC10724325; doi:10.1007/s11356-023-30945-3)
Supplement: Supplementary file 1 — Supplementary file1 (DOCX 48 KB) [file 11356_2023_30945_MOESM1_ESM.docx]

**Supplementary material**

**Exposure to caffeine over different stages of zebrafish development**

Niedja Santos^1^, Miguel Oliveira^1^ and Inês Domingues^1^

1 Department of Biology & CESAM, University of Aveiro, Campus Universitário de Santiago, 3810-193 Aveiro, Portugal

**Table S1**: Effects of caffeine (CAF) on zebrafish embryos. Statistical analysis of the swimming behaviour of fish (total distance moved; distance moved at the edges, type of movement- low, medium, and high; and path angles performed), heart rate and biochemical tests (cholinesterase; lipids; carbohydrates; proteins; oxygen consumption) in the different scenarios.

|  | **5 days exposure + recovery** | | |
| --- | --- | --- | --- |
|  | **Treatment** | **F/H value** | **p- value** |
| **Distance moved** | 5dE | 45.28 | <0.001 |
|  | 10dPE | 8.61 | 0.197 |
| **Distance moved at the edges** | 5dE | 16.91 | 0.01 |
|  | 10dPE | 1.2 | 0.321 |
| **Angles- class 1** | 5dE | 33.61 | <0.001 |
|  | 10dPE | 9.32 | 0.156 |
| **Angles- class 4** | 5dE | 24.25 | <0.001 |
|  | 10dPE | 0.555 | 0.764 |
| **Cholinesterase** | 5dE | 22.45 | <0.001 |
|  | 10dPE | 3.19 | 0.148 |
| **Hear Beat** | 5dE | 103.83 | <0.001 |
| **Lipids** | 5dE | 20.957 | <0.001 |
| **Carbohydrates** | 5dE | 22.401 | <0.001 |
| **Proteins** | 5dE | 30.89 | <0.001 |
| **Oxygen Consumption** | 5dE | 4.819 | 0.001 |
| **CEA** | 5dE | 10.802 | 0.055 |
|  | **2days exposure + recovery** | | |
|  | **Treatment** | **F/H value** | **p- value** |
| **Distance moved** | 2dE | 43.91 | <0.001 |
|  | 10dPE | 0.591 | 0.736 |
| **Distance moved at the edges** | 2dE | 3.58 | 0.003 |
|  | 10dPE | 0.295 | 0.937 |
| **Angles- class 1** | 2dE | 50.87 | <0.001 |
|  | 10dPE | 6.125 | 0.409 |
| **Angles- class 4** | 2dE | 55.59 | <0.001 |
|  | 10dPE | 6.426 | 0.377 |
| **Cholinesterase** | 2dE | 77.662 | <0.001 |
|  | 10dPE | 4.33 | 0.041 |
|  | **1 day exposure + recovery** | | |
|  | **Treatment** | **F/H value** | **p- value** |
| **Distance moved** | 1dE | 16.8 | <0.001 |
|  | 10dPE | 9.92 | 0.128 |
| **Distance moved at the edges** | 1dE | 53.74 | <0.001 |
|  | 10dPE | 5.18 | 0.521 |
| **Angles- class 1** | 1dE | 21.38 | 0.002 |
|  | 10dPE | 10.99 | 0.089 |
| **Angles- class 4** | 1dE | 27.13 | <0.001 |
|  | 10dPE | 2.065 | 0.062 |
| **Cholinesterase** | 1dE | 31.27 | <0.001 |
|  | 10dPE | 14.63 | 0.001 |
